# Editorial: Currents in biomedical signals processing—methods and applications

**DOI:** 10.3389/fnins.2022.989400

**Published:** 2022-07-29

**Authors:** Aleksandra Kawala-Sterniuk, Mariusz Pelc, Radek Martinek, Grzegorz Marcin Wójcik

**Affiliations:** ^1^Faculty of Electrical Engineering, Automatic Control and Informatics, Opole University of Technology, Opole, Poland; ^2^School of Computing and Mathematical Sciences, University of Greenwich, London, United Kingdom; ^3^Department of Cybernetics and Biomedical Engineering, Faculty of Electrical Engineering and Computer Science, VSB-Technical University of Ostrava, Ostrava, Czechia; ^4^Department of Neuroinformatics, Faculty of Mathematics, Physics and Computer Science, Institute of Computer Science, Maria Curie-Sklodowska University in Lublin, Lublin, Poland; ^5^Department of Neuroinformatics and Biomedical Engineering, Institute of Computer Science, Maria Curie-Sklodowska University, Lublin, Poland

**Keywords:** biomedical signals, signal processing, cognitive sciences, neuroscience, biomedical engineering

## 1. Introduction

Biosignals as measurement of the human body's functions provide useful information regarding human condition. Thus, the analysis of biomedical signals has become one of the most important methods for both interpretations and visualization in numerous research areas such as inter alia biology or medicine. They also play a very important role in health monitoring, diagnosis, but also as a source of data for the control purposes (in Human-Machine Interfaces). It has also led to development of numerous modern instruments designed for their detection, storage, transmission, and analysis. As the biological signals appear to be random (stochastic), it is impossible to predict their value in any time instant and therefore only statistical measures may be used in order to determine their features.

Recent advances in signal computational methods have enabled the biomedical signals in an efficient way in order to extract appropriate features and has been an interesting subject for numerous research groups for over 40 years. More and more sophisticated methods are being developed and applied including various classifiers and filters. This Research Topic should focus on the most current trends in analysis of the most popular biosignals such as EEG, ECG, EMG, which would be processed for various applications such as inter alia clinical (rehabilitation, diagnostic purposes) or control (human-machine interaction).

In this Research Topic titled “Currents in Biomedical Signals Processing—Methods and Applications” a collection of contributions showing new advancements and applications of advanced processing of biosignals for various applications was presented.

The paper Binkowska et al. was focused on association between long-term cannabis use and memory impairments. Based on authors' thorough literature review—very few studies have examined this relationship, in particular in a poly-drug context, when cannabis was combined with other addictive substances. For this purpose the authors used event-related potentials for the examination of the recognition process in a visual episodic memory task in both cannabis users (CU) and cannabis poly-drug users (PU). The hypothesis stated that both groups (CU and PU) will have their behavioral and phychophysiological-indicators of memory processes affected with the use of cannabis alone or with other substances, with the PU patients expressing stronger changes. The obtained results proved alterations in recognition memory processing in both groups compared to the healthy participants from the control group (CG), and these were at most visible in cannabis poly-drug users (PU), while there was no significant effect for patients using cannabis alone. The paper proves that the use of cannabis with other substances results with the biggest disturbance in the brain function. The authors applied analysis of electroencephalography (EEG) signals for this study, which is non-invasive.

Another study involving analysis of EEG signals was presented in Kwasniewicz et al., where the authors used brain signals for the purpose of message credibility evaluation. No similar studies could be found in the literature and the efficiency of the obtained results was over 0.7. The authors found out appropriate brain areas active depending on positive or negative message reliability.

Also in Jakubowska et al., the authors used EEG signals, where they assessed visual working memory (VWM) capacity. The authors tired to assess the efficiency of training conducted on a Real-Time Strategy (RTS) video game (StarCraft II) for the purpose of VWM capacity improvement. The study involved 62 participants, who took part in two EEG sessions (before and after the StarCraft II-based training).

Analysis of the EEG signals, which is a very challenging task mostly due to the nature of these signals, was also presented in paper Xu et al.; where the authors applied topological data analysis (TDA) in order to analyse and understand the brain signals. This paper is a short review and shows the opportunities in analysis of EEG signals brought by the TDA, which differs from traditional signal processing methods and has a very wise potential usage in among the others Brain-Computer Interfaces (BCIs).

As mentioned above—analysis of the EEG signals is difficult among the others due to these signals' non-stationarity. Also the electroencephalography signals are prone to various external and internal artifacts occurrence, where the internal artifacts are frequently related with physiological activity, such as the one related with eye movement and blinking. The eye blinking (EB) artifacts strongly affect the signals' quality. In Jurczak et al., the authors compared the most popular methods applied for the electrooculography (EOG) artifacts removal—independent component analysis (ICA) and regression with the convolutional neural network (CNN) in order to eliminate the eye blinking artifacts. The obtained results showed much higher performance of the CNN method than the one given by regression or ICA.

In Barry et al. the authors focused on using more invasive brain signals—electrocorticography (ECoG). The main aim of this work was to examine ECoG spectrogram images for training proper and reliable cross-patient seizure classifiers. In this paper, the data collected from 113 patients was converted into RGB spectrogram images. For classification purposes five different convolutional neural networks (CNN) were applied, which gave the cross-patient classification (seizure/non-seizure) accuracy of 87.9%, while the appropriate trained ResnEt50-based models provided efficiency of 95.7%, which is very high.

Another study using deep learning algorithms such as convolutional neural networks, in this case the osteogenic convolutional neural network (OCNN), was presented in Lan et al., where the authors decided to use them for the rat bone marrow mesenchymal stem cells (rBMSCs) osteogenic differentiation quantitative measurement. The study showed that the OCNN enabled successful distinguishing of differentiated cells at a very early stage and gave better prediction performance compared with the single morphological parameters. Based on the conducted experiments it is possible apply the OCNN-based online learning models for further rBMSCs osteogenic differentiation recognition.

The authors of Haraguchi et al. described the use of less common signals, namely magnetoencephalography (MEG), where the effect of menstrual cycle on brain activity has been analyzed. In this work, the authors focused on objective quantitative MEG parameters in order to investigate the effects of the menstrual cycle on spontaneous neural oscillations. The study involved 25 healthy female participant, with normal, regular menstrual cycle. The testing was conducted twice: during menstrual period (MP) and outside period (OP). The authors of this paper showed that it is possible to use the menstrual cycle as an accurate interpretation of functional neuroimaging in clinical practice.

In Bartosik et al. statistical analysis was applied for the purpose of selecting the most important personality features based on attractiveness assessment. Based on authors thorough literature study and on their research experience they claimed that trust is based on facial appearance appraisal, which is made based on facial morphological characteristics, such as among the others color, complexion, shape, etc. In order to select appropriate features the authors modeled a backward step-wise logistic regression; they also analyzed the results of the psychological tests together with the attractiveness and trust survey.

In paper Boschen et al., the authors presented Fast Scan Cyclic Voltammetry (FSCV), which has been used for many years in animal models, but has not been applied on humans. The main aim of this work was to bring interest into using the FSCV in human clinical studies. This article showed also some technical challenges, which may limit its clinical implementation, but these can be overcome while properly addressed.

As mentioned above—analysis of biomedical data is a very challenging task, but this makes is very interesting. We hope that our Research Topic will be found interesting to readers and researchers in fields of medicine, biomedical engineering or neuroscience.

## Author contributions

All authors reviewed and accepted the manuscript for submission.

## Conflict of interest

The authors declare that the research was conducted in the absence of any commercial or financial relationships that could be construed as a potential conflict of interest.

